# Landscape drivers of recent fire activity (2001-2017) in south-central Chile

**DOI:** 10.1371/journal.pone.0201195

**Published:** 2018-08-22

**Authors:** David B. McWethy, Aníbal Pauchard, Rafael A. García, Andrés Holz, Mauro E. González, Thomas T. Veblen, Julian Stahl, Bryce Currey

**Affiliations:** 1 Department of Earth Sciences, Montana State University, Bozeman, Montana, United States of America; 2 Laboratorio de Invasiones Biológicas, Facultad de Ciencias Forestales, Universidad de Concepción, Concepción, Chile; 3 Instituto de Ecología y Biodiversidad (IEB), Santiago, Chile; 4 Department of Geography, Portland State University, Portland, Oregon, United States of America; 5 Facultad de Ciencias Forestales y Recursos Naturales, Instituto de Conservación, Biodiversidad y Territorio, Laboratorio de Ecología de Bosques, Universidad Austral de Chile, Valdivia, Chile; 6 Center for Climate and Resilience Research (CR)^2^, Santiago, Chile; 7 Department of Geography, University of Colorado, Boulder, Colorado, United States of America; 8 Department of Land Resources and Environmental Sciences, Montana State University, Bozeman, Montana, United States of America; Pacific Northwest National Laboratory, UNITED STATES

## Abstract

In recent decades large fires have affected communities throughout central and southern Chile with great social and ecological consequences. Despite this high fire activity, the controls and drivers and the spatiotemporal pattern of fires are not well understood. To identify the large-scale trends and drivers of recent fire activity across six regions in south-central Chile (~32–40° S Latitude) we evaluated MODIS satellite-derived fire detections and compared this data with Chilean Forest Service records for the period 2001–2017. MODIS burned area estimates provide a spatially and temporally comprehensive record of fire activity across an important bioclimatic transition zone between dry Mediterranean shrublands/sclerophyllous forests and wetter deciduous-broadleaf evergreen forests. Results suggest fire activity was highly variable in any given year, with no statistically significant trend in the number of fires or mean annual area burned. Evaluation of the variables associated with spatiotemporal patterns of fire for the 2001–2017 period indicate vegetation type, biophysical conditions (e.g., elevation, slope), mean annual and seasonal climatic conditions (e.g., precipitation) and mean population density have the greatest influence on the probability of fire occurrence and burned area for any given year. Both the number of fires and annual area burned were greatest in warmer, biomass-rich lowland Bío-Bío and Araucanía regions. Resource selection analyses indicate fire ‘preferentially’ occurs in exotic plantation forests, mixed native-exotic forests, native sclerophyll forests, pasture lands and matorral, vegetation types that all provide abundant, flammable and connected biomass for burning. Structurally and compositionally homogenous exotic plantation forests may promote fire spread greater than native deciduous-Nothofagaceae forests which were once widespread in the southern parts of the study area. In the future, the coincidence of warmer and drier conditions in landscapes dominated by flammable and fuel-rich forest plantations and mixed native-exotic and sclerophyll forests are likely to further promote large fires in south-central Chile.

## Introduction

In recent decades, surges in wildfire activity in many ecosystems worldwide, even where fuel conditions and natural ignitions historically limited fire activity, have prompted questions of whether climate change, human ignitions, land-use change, and/or altered vegetation are responsible [1, 2]. Many of the largest fires are occurring where highly flammable exotic vegetation and homogeneous forest plantations have replaced more heterogeneous and less fire-prone native vegetation [3, 4]. At the same time, extended drought, warming temperatures and rapid land-use change are compounding alterations in fuel conditions that promote fire [5–8]. Temperate and Mediterranean forests have recently experienced large and destructive fires that may signal new norms in future conditions [9]. Anomalously large fire years in these settings have been attributed to drier-than-average summers, prolonged drought and longer fire seasons as well as increased quantities of fine fuel lagging wet years, raising concerns about the trajectory of post-fire vegetation dynamics and future fire regimes [6, 10].

In south-central Chile (32–40° S. Latitude), Mediterranean shrublands and sclerophyllous forests co-occur with temperate deciduous forests. Here, abundant vegetative biomass and seasonal desiccation support some of the highest levels of fire activity in South America [11, 12]. Recent fires in Valparaíso, Santiago and cities and villages throughout the Bío-Bío and Araucanía regions illustrate the risks and hazards of fires to communities and ecosystems [13]. The impacts have prompted the Chilean President and the Ministry of the Environment to call for an investigation of the factors responsible. During the fire season of 2016–2017 over 580,000 hectares burned in central Chile [14], the largest extent of area burned recorded in Chile since detailed records started in the early 1960s. The large fires of 2016–2017 have stimulated communities and government agencies to ask what land-use policies and environmental factors are responsible for these large fire events, generating a national debate about strategies that could be developed to prevent and mitigate the negative consequences of future large fires [14–16].

In recent decades, structurally and compositionally homogeneous industrial plantations (*Eucalyptus* and *Pinus* spp.) have expanded throughout south-central Chile in areas previously occupied by a more heterogeneous matrix of native forests, agricultural lands and degraded open forest/shrublands [8]. Coupled with warming temperatures and drought, the increase in the continuity of flammable woody fuels associated with the expansion of plantation forests is thought to be shifting the underlying conditions that mediate fire activity. This has been shown to occur elsewhere [2, 9, 17], especially along the transition between Mediterranean and Temperate bioclimatic zones. Additionally, previous research suggests that the overall flammability and fuel loads are higher in areas where invasive plant species have invaded native forests [18–21]. The regeneration of some invasive plant species is favored by fire, resulting in a positive feedback between invasive plant abundance and fire activity [22–24]. Invasive plants have the capability of altering the resilience of native vegetation by modifying the predominant regeneration strategy following fire and post-fire traits (e.g., from seeders to resprouters; [25–27]). Thus, land cover change to more fire-prone exotic industrial plantations and climatic conditions may be contributing to increased fire activity in south-central Chile [12, 13, 28, 29]. It is therefore timely to evaluate whether changing climate and land-cover conditions are altering the drivers and probability of fire occurrence along this Mediterranean-Temperate boundary. These include changes in the spatial distribution and heterogeneity of vegetation types that promote or inhibit fire, most importantly those associated with plantations of exotic trees and pasture and agricultural lands.

**Fig 1:**
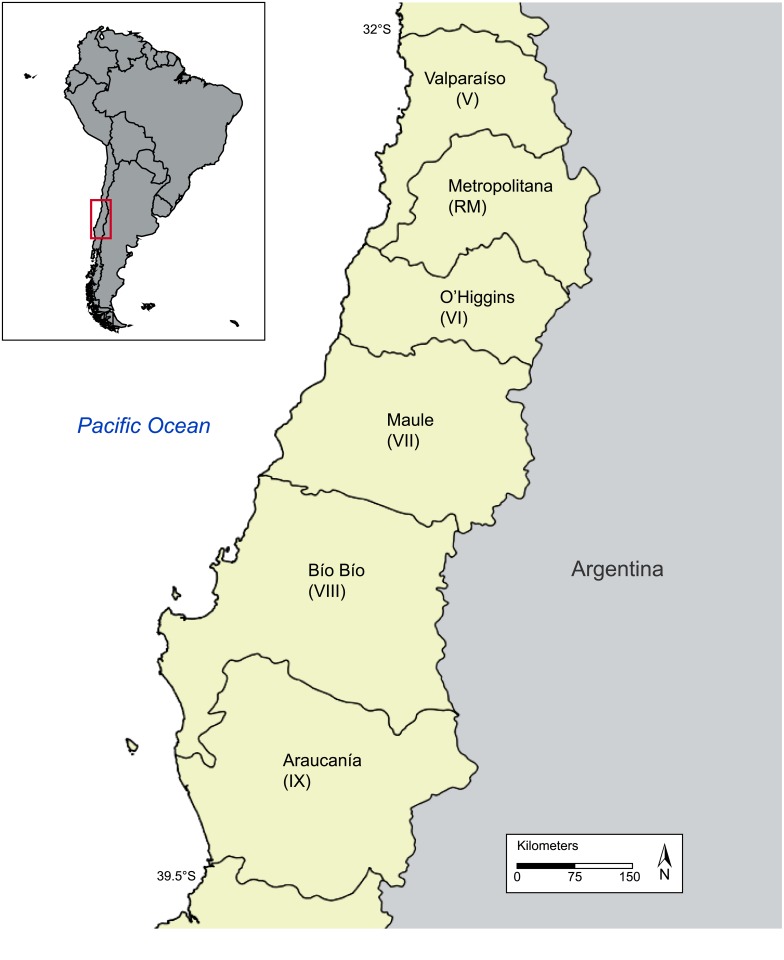
Study area. Administrative regions of south-central Chile included in this study.

**Fig 2:**
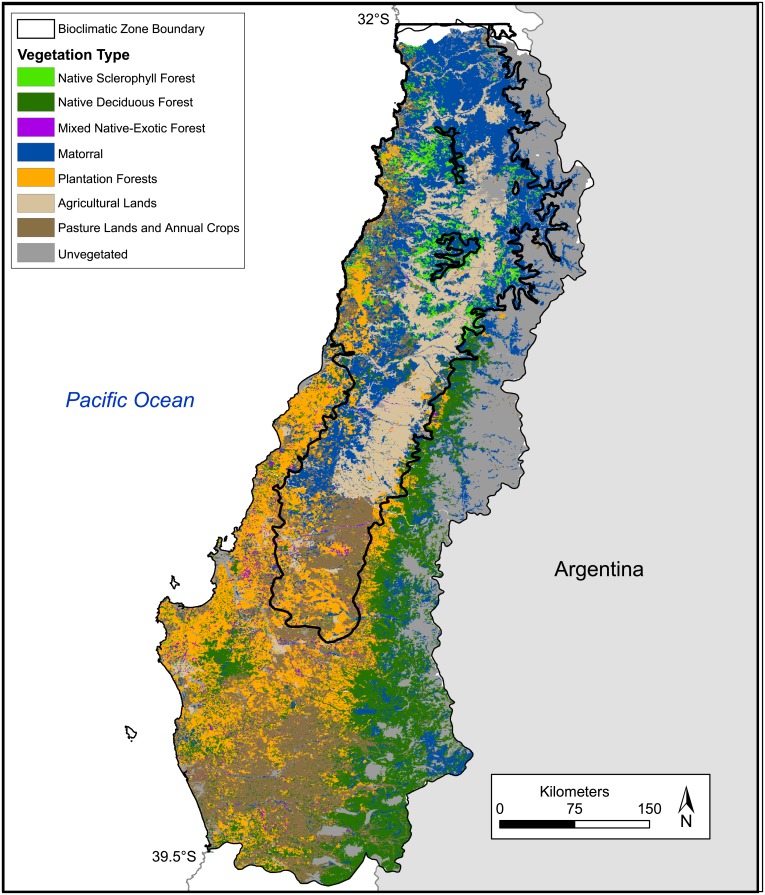
Vegetation types. Vegetation types derived from Corporación Nacional Forestal of Chile Catastro database [31] and bioclimatic zone boundaries adapted from Gajardo [32] and Veblen et al. [33] (solid black line) separating the Mediterranean bioclimatic zone and the Temperate bioclimatic zone.

Here we set out to investigate two key questions: what is the relationship between the spatial distribution of recent fire activity in south-central Chile and variables known to influence fire activity? What landscapes are most vulnerable to fire occurrence now and into the future? The central objectives of this paper are to: 1) characterize the spatial and temporal trends of satellite-derived fire activity for two bioclimatic zones in south-central Chile, 2) identify the key variables that best predict spatiotemporal patterns of recent fires in these bioclimatic zones, 3) map the modeled spatial variability of fire probability across the study area, and 4) evaluate whether fire occurrence indicates selective ‘preference’ for specific vegetation types. To accomplish these objectives, we evaluate a satellite-derived burned area dataset for the period 2001–2017 in six administrative regions of south-central Chile (Fig 1, Table 1) from Moderate Resolution Imaging Spectroradiometer (MODIS) imagery. First, we characterize burned area from MODIS Collection 6 burned area data (MCD64A1, [30]) and compare with Corporación Nacional Forestal of Chile (CONAF) fire records. Second, we use generalized linear, generalized additive and classification and decision tree models to evaluate relationships between fire occurrence and climatic, fuel (based on 8 general vegetation types shown in Fig 2), and human factors known to influence fire activity. Third, we use model results to map the probability of fire occurrence across south-central Chile.

**Table 1:** Administrative regions of south-central Chile included in this study.

Administrative Region	Region Code	Area (km^2^)	Bioclimatic Zone
Valparaíso	V	16,396	Mediterranean
Metropolitana	RM	15,403	Mediterranean
O’Higgins	VI	16,387	Mediterranean
Maule	VII	30,296	Mediterranean/Temperate
Bío-Bío	VIII	37,069	Mediterranean/Temperate
Araucanía	IX	31,842	Temperate

## Materials and methods

### Study area

The study area spans ~32–40° S. latitude, including six administrative regions (hereafter “regions”) from Valparaíso to La Araucańia (Fig 1). The drier northern bioclimatic zone (between approximately 32–36° S) supports Mediterranean-matorral shrub and sclerophyllous forests whereas the wetter southern bioclimatic zone supports temperate deciduous and broadleaf evergreen forests (between approximately 36–40° S, Fig 2; [32–34]). These two bioclimatic zones span a critical transition between dry, shrubland (fuel-limited) fire regimes in the north to wetter, climate-limited fire regimes in the south. We focus on these zones because we expect them to be most vulnerable to increasing fire hazard related to changes in both climate and land-cover, and because they include the highest population densities found in Chile. The two bioclimatic zones, hereafter referred to as the “North” and “South” bioclimatic zones, encompass highly productive coastal forests (>1000 gCm^2^/yr), unproductive (<100 gCm^2^/yr) arid shrublands and high alpine ecosystems (Fig 2) [35]. The primary controls on the distribution, abundance and structure of vegetation varies from limited water availability and high temperatures in the North bioclimatic zone to limited availability of solar radiation and cool temperatures in the South bioclimatic zone [32, 34].

In south-central Chile the dominant geographic controls on the distribution of vegetation include elevational gradients associated with north to south mountain ranges including the coastal cordillera and the Andes and east-west ranges, tectonically and erosionally created lowlands and valleys (e.g., central valley including the Maipo and Maule valleys), seasonal temperatures and the distribution of precipitation [36]. Additionally, local topography, substrate and microclimate act as fine-scale controls. The topographically and ecologically heterogeneous North bioclimatic zone experiences warm, dry summer conditions and moderate winter temperatures with a single, short rainy season [37]. This zone is dominated by evergreen matorral shrublands and sclerophyllous woodland (bosque esclerófilo) in lowlands and foothills. These communities—according to altitude and topography (south- or north facing slopes)—exhibit mixed dominance of many shrub species and common trees such as *Lithraea caustica*, *Peumus boldus*, *Quillaja saponaria*, *Cryptocarya alba*, *Beilschmiedia miersii*. An open savanna community known as espinal (*Acacia caven*) cover the low-flat areas of the central valley [38]. Moreover, deciduous forests (e.g., *Nothofagus spp*.) in more mesic areas (south-facing slopes) and steppe vegetation occur at higher elevations [38–40]. The South bioclimatic zone experiences moderate annual temperatures, warm summers and cool winters with most precipitation occurring during winter months. Persistent westerly flow and associated moisture delivery supports extensive temperate deciduous evergreen forests that are referred to as the mixed deciduous-evergreen temperate forests [32, 33, 41]. The northern portion of this wetter zone is dominated by deciduous/evergreen *Nothofagus* forests (*Nothofagus obliqua*, *N*. *nervosa*, *N*. *dombeyi*) which are mixed at different altitudes with broadleaf evergreen taxa (e.g., *Laureliopsis philippiana*, *Aextoxicon punctatum*, *Eucryphia cordifolia*, *Weinmannia trichosperma*, *Drimys winteri*, *Myrtacea*) and coniferous podocarps [42]. *Araucaria araucana*, a long-lived conifer is the dominant Andean forest (above 1000 masl) in the Araucanía region, pure or mixed with *N*. *dombeyi*, *N*. *pumilio* or *N*. *antarctica* [43]

### Controls on wildfire

To identify the primary controls on fire activity across the large spatial area of south-central Chile we evaluated a number of local to regional scale variables including variables that operate at finer spatial resolution than those considered by Holz et al. [44]. These include variables representing climatic conditions that influence the flammability (temperature, precipitation), spatial distribution, continuity and abundance of fuels (land cover) and the distribution of natural and human-caused ignitions (lightning flash rate, population density) (Table 2). Based on previous research by Holz et al. [44] and by acknowledging major differences in primary productivity vegetation cover and climatic conditions, we evaluated drivers of fire activity separately for the dry North bioclimatic zone and the wetter South bioclimatic zone.

**Table 2:** Variables used in the initial model analyses. Variables included in the final model selection are discussed in text. All data were resampled to 500m^2^.

Predictor Category and Variable	Data Resolution	Data Source
***Climate***		
Mean annual temperature (C°)	30 arc seconds	Fick & Hijmans 2017 (worldclim2.4)
Total annual rainfall (mm)	30 arc seconds	Fick & Hijmans 2017 (worldclim2.4)
Mean spring rainfall (Sep-Nov, mm)	30 arc seconds	Fick & Hijmans 2017 (worldclim2.4)
Mean growing season rainfall (Oct-March, mm)	30 arc seconds	Fick & Hijmans 2017 (worldclim2.4)
Mean autumn rainfall (April-June, mm)	30 arc seconds	Fick & Hijmans 2017 (worldclim2.4)
***Topography***		
Elevation (masl)	30 m	NASA ASTER GDEM (asterweb.jpl.nasa.gov)
Northness index (slope/aspect interaction)	30 m	NASA ASTER GDEM (asterweb.jpl.nasa.gov)
Slope (°)	30 m	NASA ASTER GDEM (asterweb.jpl.nasa.gov)
***Land Cover***		
Vegetation type (8 types)	0.06 km^2^	CONAF (http://www.conaf.cl/)
Invasive spp. index (# spp. per 10km^2^)	10 km^2^	Fuentes et al. 2013
***Ignitions***		
Annual lightning flash rate (strikes km^2^ yr^-1^)	1 km^2^	Cecil et al. 2014
***Human activity***		
Mean population density (inhabitants km^2^)	5-arc-minute	HYDE dataset, Klein Goldewijk et al. 2016

#### Fire dataset

Historically, wildfire occurrence in south-central Chile was most common during the warm and dry summer season (January-March) although fires in recent decades have been recorded throughout the year in many parts of the study area [12, 14]. The fire season was defined according to a protocol used by CONAF which records fire incidents in annual periods from July 1^st^ to June 30^th^. Hence, the fire year 2001–2002 represents fire activity beginning July 1^st^, 2001 and ending June 30^th^, 2002. The Bío-Bío and Araucanía regions experience the highest number of fires annually [45]. For CONAF records, fires are detected via ground, aerial and satellite approaches (http://www.conaf.cl/) and fire detections primarily rely on the presence and spatial distribution of mobile and fixed ground and aerial operators which are complemented by satellite derived fire detections. CONAF fire data includes forested areas and large portions of other vegetation types but does not record prescribed fires in agricultural and pasture lands. Here we focus our analyses on satellite-derived fire detections which provide comprehensive spatial and temporal coverage across all forested and non-forest lands and validation of fire detections with a time-series of satellite derived surface reflectance values. We also compare satellite derived versus CONAF burned area estimates to evaluate fire activity recorded by the different approaches.

**Fig 3:**
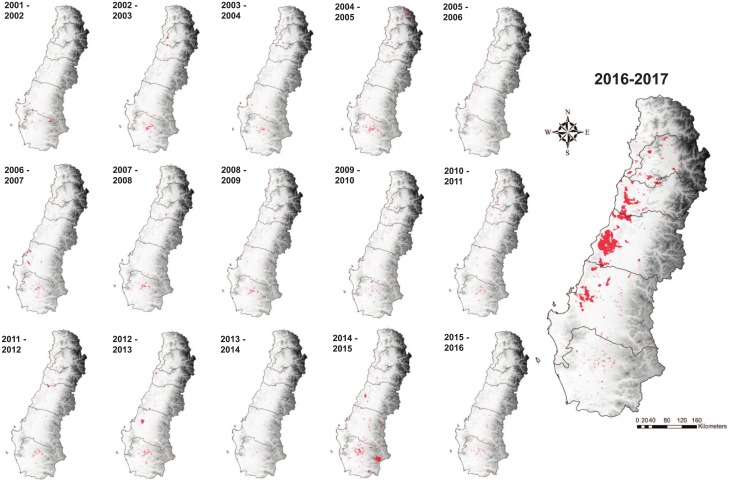
Spatial distribution of burned area for all fire years 2001–2017 (MODIS Collection 6 burned area detections). Burned areas within each fire year indicated with red shading.

**Fig 4:**
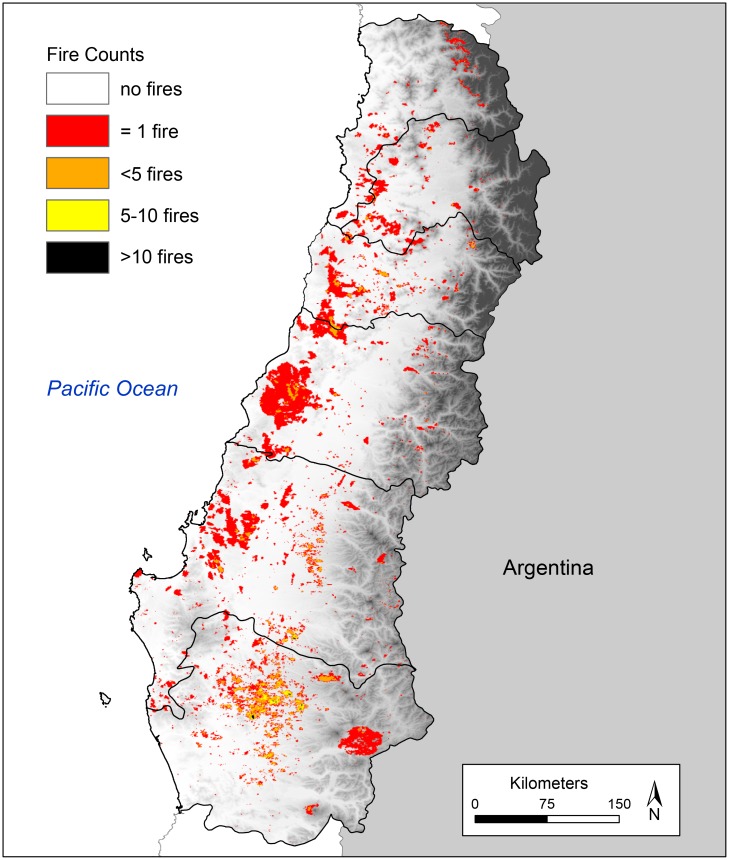
Spatial distribution of burned area by number of fire counts per raster cell (each raster cell = 21.47 hectares) for all fire years 2001–2017 (MODIS Collection 6 burned area detections).

We used fire detections from satellite derived MODIS Collection 6 burned area product (MCD64A1, [30]) obtained from MODIS Aqua and Terra data imagery (Figs 3 and 4). The MODIS Collection 6 product is a daily gridded product for each 500 m^2^ burned pixel. We used the MODIS Collection 6 burned area product to first, characterize fire activity over the 2001–2017 interval, second, to identify key drivers of the spatial distribution of burned area in the six central Chilean regions and third, to model the probability of fire occurrence across the study area using generalized linear, generalized additive and decision tree models. The MODIS Collection 6 product uses a synergistic approach that includes both abrupt changes in surface reflection and information from 1km MODIS active fire data [30, 46]. The MODIS Collection 6 burned area product detects significantly more burned area than the previous Collection 5.1 MCD45A1 and MCD64A1 burned area products [46]. As a result of MODIS tile extent, fire data for the far northwestern corner of the Valparaiso region was not included in our analyses.

#### Evaluating important drivers of fire activity

We used generalized linear models (GLMs, [47]), generalized additive models (GAMs, [47]) and the decision tree classification algorithm Random Forests [48] to model the probability of fire occurrence across the study area. We used R-package ‘mgcv’ [49] to evaluate the relationship between individual predictors and the occurrence of fire and the Akaike Information Criterion (AIC) comparison to avoid model overfitting [50]. We also employed variable screening for binary classification models using information theory and Random Forests ‘variable importance’ estimates for all variables prior to being selected for GLM and GAM analyses (S1 Table). Weight of Evidence (WOE), Information Value (IV) [51–53] and Variable Importance (VI) estimates from Random Forests [48] all provide pre-screening of variables by assessing univariate predictive strength and help prevent overfitting. After pre-screening of variables using a comparison of WOE, IV and VI values, highly collinear variables (r > 0.60) from the complete set of predictor variables (Table 2) were removed prior to GLM and GAM analyses.

The multi-model approach used here provides complementary information useful for better understanding the relationship between fire activity and predictor values. GLMs estimate univariate relationships between factors known to influence fire activity (e.g., climate, fuel conditions and ignitions) yet this approach assumes the relationship between the response and predictor variables is linear. Generalized additive models are more flexible, allowing the relationship to be determined by the data but can often result in overfitting. Random Forests decision tree models are less sensitive to correlated variables, have strong predictive power and estimate the importance of each variable in contributing to model performance [54].

In addition to identifying models that best predict the spatial distribution of fire occurrence, we employed resource selection functions to determine whether the probability of fire occurrence in each vegetation type was higher or lower than expected based on the availability of each vegetation type. We used the Murdoch’s Index and the R-package ‘Resource Selection Function’ [49, 55–57] to assess selective preference. The Murdoch’s Index of resource selection is preferred because it provides symmetrical preference estimates and has the desirable attribute that the availability of resources (i.e., vegetation type) does not affect the preference value [58].

## Results

### Trends in burned area 2001–2017

**Fig 5:**
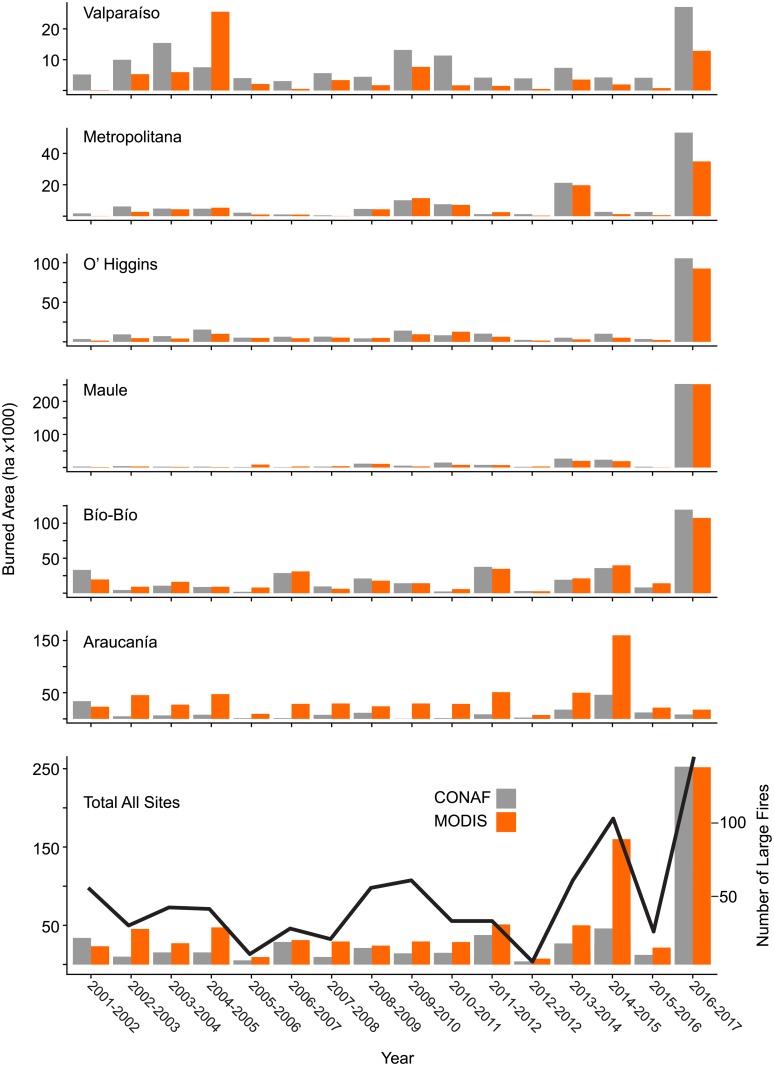
Total burned are for each region by year. Burned area by year across administrative regions based on CONAF dataset (gray bars) and MODIS Collection 6 burned area detections (orange bars; Y-axis scale varies for each region). Total burned area for all districts based on CONAF dataset and MODIS detected burned area (bottom panel). Total number of large fires (>200 ha) by year from CONAF dataset indicated by black line.

**Fig 6:**
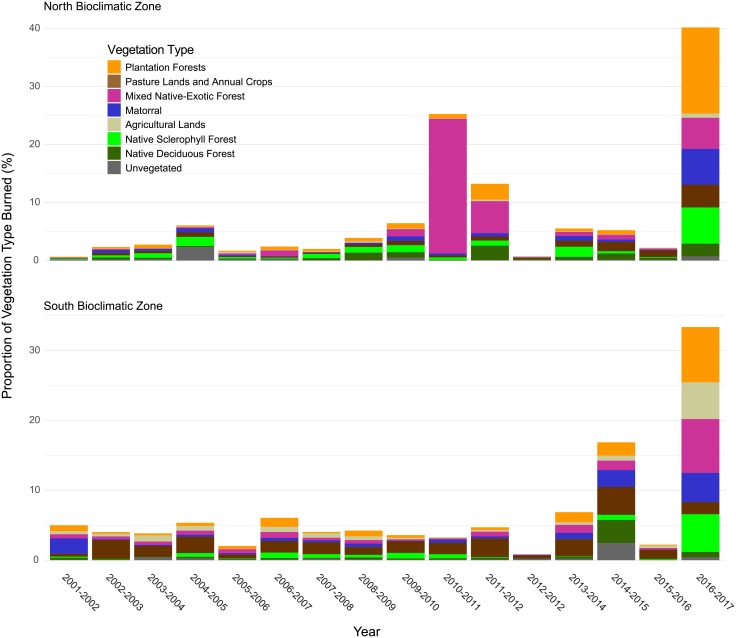
Proportion of each vegetation type burned by year. Proportion of each land cover/vegetation type burned each year for the North bioclimatic zone (top panel) and South bioclimatic zone (bottom panel) (MODIS Collection 6 burned area detections).

**Fig 7:**
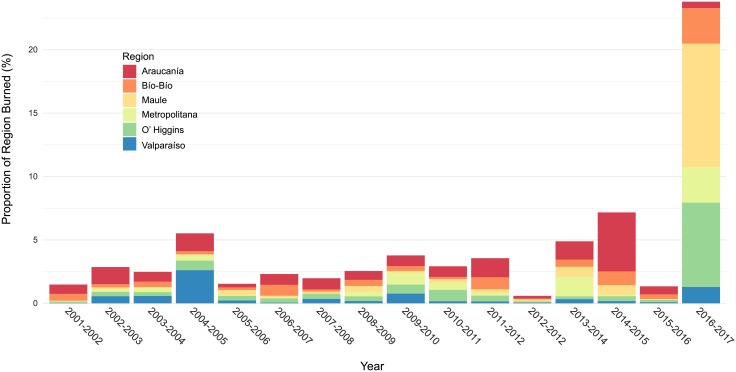
Proportion of each region burned by year. Proportion of each region burned each year (MODIS Collection 6 burned area detections).

CONAF and MODIS satellite-derived fire records indicate strong year-to-year variability in both the frequency of fires and total area burned and no significant trends over time (Fig 5). MODIS data recorded a mean annual burned area of 103,169 ha/yr for the entire study area compared to CONAF burned area estimates which recorded mean annual burned area of 85,707 ha/yr (13.60 million ha total burnable area). The North and South bioclimatic zones recorded mean annual burned areas of 33,793 ha/yr (5.6 million ha total burnable area) and 44,738 ha/yr (7.53 million ha total burnable area) respectively. Across the drier North bioclimatic zone, MODIS satellite-derived burned area as a proportion of mean total area burned each year within this zone was greatest in matorral, pasture lands and plantation forests (Fig 6, Table 3). Across the more mesic South bioclimatic zone the proportion of burned area was greatest in exotic plantation forests, pasture lands and annual crops, native deciduous forests and matorral. Mean annual and total area burned (2001–2017) was greatest in the Araucanía, Bío-Bío, and Maule regions (Fig 7, Table 4).

**Table 3:** Mean annual and total burned area (2001–2017) in hectares (ha) by vegetation type for the North and South bioclimatic zones (MODIS Collection 6 burned area detections).

NORTH BIOCLIMATIC ZONE			
**Vegetation Type**	**Mean Annual Burned Area (ha)**	**Total Burned Area (ha)**	**Total Area of Vegetation Type (ha)**
Native Sclerophyll Forest	2,863	45,808	302,947
Native Deciduous Forest	460	7,363	78,543
Mixed Native-Exotic Forest	333	5,324	36,127
Matorral	13,841	221,463	2,048,310
Plantation Forest	8,164	130,620	616,970
Pasture Lands and Annual Crops	4,883	78,136	795,437
Agricultural Lands	1,988	31,812	1,311,260
Unvegetated	1,174	18,783	415,363
SOUTH BIOCLIMATIC ZONE			
**Vegetation Type**	**Mean Annual Burned Area (ha)**	**Total Burned Area (ha)**	**Total Area of Vegetation Type (ha)**
Native Sclerophyll Forest	254	4,057	40,957
Native Deciduous Forest	7,026	112,417	2,061,940
Mixed Native-Exotic Forest	958	15,327	114,885
Matorral	4,948	79,166	741,214
Plantation Forest	17,005	272,080	1,768,159
Pasture Lands and Annual Crops	12,343	197,486	1,634,792
Agricultural Lands	649	10,389	111,000
Unvegetated	1,546	24,729	636,633

**Table 4:** Mean annual and total burned area (ha) by administrative region (MODIS Collection 6 burned area detections).

Administrative Region (Region Code)	Mean Annual Burned Area	Total Burned Area (2001–2017)
Valparaíso (V)	4,694	75,109
Metropolitana (RM)	6,067	97,069
O’Higgins (VI)	10,859	173,745
Maule (VII)	21,689	347,017
Bío Bío (VIII)	22,341	357,450
Araucanía (IX)	37,520	600,314

Fires burned a higher proportion of the Araucanía region compared to other regions except for the 2016–2017 fire season when large percentages of the Maule, O’Higgins, Bío-Bío and Metropolitana regions burned (Fig 7). Annual burned area estimates derived from MODIS Collection 6 satellite fire detections were consistently higher than CONAF burned area records for the Araucanía region (Fig 5). MODIS total annual burned area estimates for all six south-central regions was also higher than CONAF fire burned area records for most years, largely as a result of higher annual burned area estimates for the Araucanía region where extensive use of prescribed burns in pasture lands and annual crops are not reflected in CONAF records.

### Model selection

Results from pre-screening of model variables and generalized linear and generalized additive model selection indicate elevation, growing season precipitation, population density, slope and vegetation type best explain the probability of fire occurrence in south-central Chile (S1–S3 Tables). Variable importance values from Random Forests models identify population density, mean growing season precipitation and slope as the most important variables contributing to the network of decision trees best predicting the probability of fire occurrence (Table 5). The deviance explained by the best GAMs for the entire study area (12.4%) and the North (12.6%) and South (14.9%) bioclimatic zones was generally low. The receiver operating characteristic curve (ROC) area under the curve (AUC) model performance estimates based on modeled and training datasets were higher for the Random Forests models (entire study area = 0.950, North bioclimatic zone = 0.948, South bioclimatic zone = 0.955) than the AUC model estimates for the best GAMs (entire study area = 0.734, North bioclimatic zone = 0.761, South bioclimatic zone = 0.765, Table 5).

**Table 5:** Comparison of best GAM variable coefficients for entire study area and the North and South bioclimatic zones. Variable coefficients (Coef), standard errors (SE) shown for categorical variable vegetation and estimated degrees of freedom (edf) for continuous variables. The receiver operating characteristic area under the curve (ROC AUC) value for each model is shown in parentheses.

Best GAM	Study Area (0.734)	North BZ (0.761)	South BZ (0.765)
Variable	Coef/SE	Coef/SE	Coef/SE
Native Sclerophyll Forest	-56.032 ± 10.175	-43.944 ± 8.287	-2.796 ± 0.086
Native Deciduous Forest	-0.287 ± 0.092	-0.578 ± 0.064	-0.059 ± 0.084
Mixed Native-Exotic Forest	-0.116 ± 0.147	0.229 ± 0.077	0.276 ± 0.093
Matorral	-0.197 ± 0.079	-0.2 ± 0.028	0.316 ± 0.083
Plantation Forest	0.174 ± 0.084	0.29 ± 0.032	0.345 ± 0.083
Pasture Lands and Annual Crops	-0.037 ± 0.087	-0.394 ± 0.033	0.409 ± 0.084
Agricultural Lands	-1.858 ± 0.107	-1.503 ± 0.039	0.025 ± 0.097
Unvegetated	-0.865 ± 0.109	-0.725 ± 0.048	-0.151 ± 0.091
	**edf**	**edf**	**edf**
Elevation	8.994	8.942	8.844
Grow Seas. Precip.	7.763	8.996	8.975
Population Density	8.416	8.856	7.758
Slope	7.976	8.345	8.566

**Fig 8:**
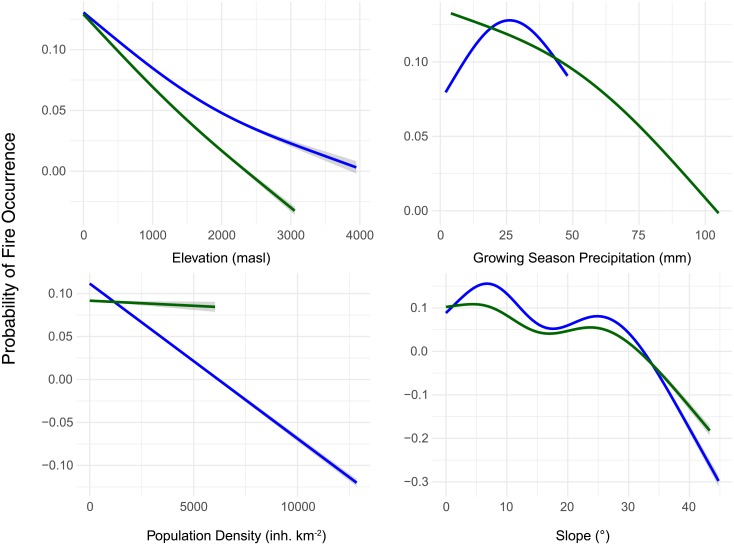
Univariate relationship between the probability of fire occurrence and continuous variables included in best GLM, GAM and Random Forests models. Plots represent predicted relationship between the probability of fire occurrence and univariate predictors for the North (blue lines) and South (green lines) bioclimatic zones.

The relationship between univariate biophysical, climatic and population density variables and the probability of fire occurrence suggests that the probability of fire decreases as elevation increases (Fig 8). The probability of fire occurrence declines with increasing precipitation in the South bioclimatic zone and exhibits a unimodel relationship with mean growing season precipitation in the North bioclimatic zone. The probability of fire occurrence generally decreases with increased population density in the North bioclimatic zone, but this relationship is weaker in the South bioclimatic zone (Fig 8).

**Fig 9:**
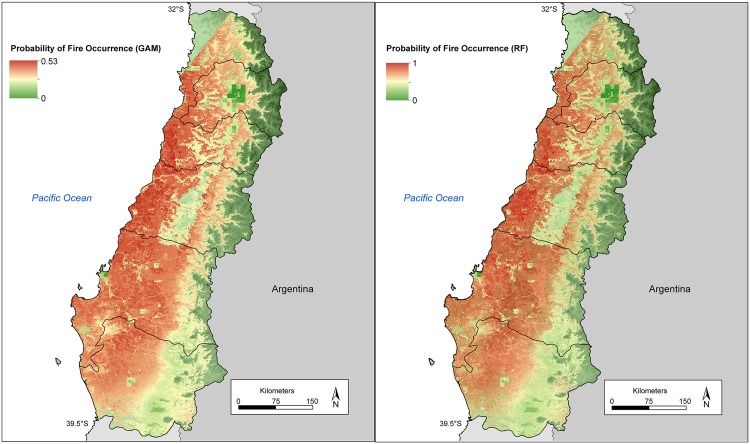
Spatial distribution of the probability of fire occurrence based on best GAM and Random Forests models.

The probability of fire occurrence estimated by the best GLM, GAM and Random Forests models was greatest in coastal mountain matorral and plantation forests in the North bioclimatic zone and coastal plantation forests, pasture lands and lowest in agricultural lands, higher elevations and the far northern matorral shrublands (Fig 9, see S1 Fig for GLM model results). In the South bioclimatic zone, the probability of fire occurrence was greatest in plantation forests and pasture lands and annual crops concentrated in lowland valleys and lowest in native deciduous forests, at higher elevations and the wetter far southern extent of the South bioclimatic zone (Fig 9, S1 Fig).

**Fig 10:**
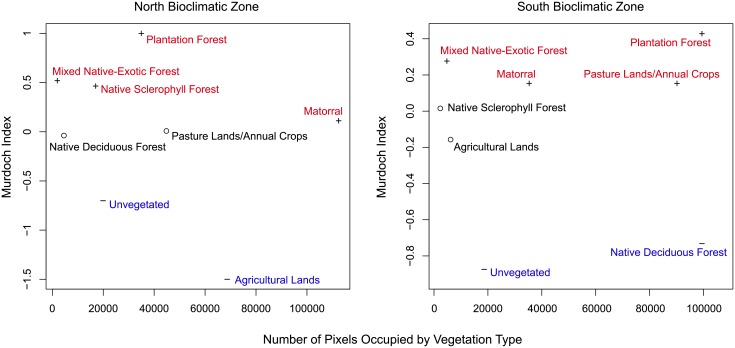
Fire selective ‘preference’ for specific vegetation types based on the Murdoch’s Index [58].

Results from resource selection function analyses for the North bioclimatic zone show strong fire preference for plantation forests, mixed native-exotic forests, native sclerophyll forests, and matorral, and show avoidance in agriculture and unvegetated lands (land cover primarily comprised of rock, ice, snow, beaches, dunes, lakes, and rivers; Fig 10, S2 Fig). In the South bioclimatic zone, results indicate fire preference for plantation forests, pasture lands and annual crops, mixed native-exotic forests, and matorral and avoidance in native deciduous forests and unvegetated lands (Fig 10, S2 Fig).

## Discussion

Consistent with the varying constraints model, fire records from south-central Chile highlight a broad zone experiencing high mean annual fire activity at intermediate locations in the Mediterranean-type sclerophyll ecosystems and adjacent transitional temperate deciduous forests where resources to burn are abundant in all years and fuel moisture dries under reliably dry summer conditions. Our results parallel those reported by Holz et al. [44, 59] and Urrutia et al. [59] who found that low net primary productivity in the northern part of our study area limited fuel availability despite seasonal drying, whereas abundant fuels at the southern end of their study area rarely dried enough to promote fire spread facilitating large fire events. Across the entire study area MODIS satellite detections show similar spatiotemporal trends in fire activity recorded by CONAF records but do not register large (mostly prescribed) fires that occur each year in pasture lands and annual crops, especially in the Araucanía region. Over the 2001–2017 period, spatiotemporal patterns in area burned show no trend suggesting that year-to-year variability in climate and fuel conditions influences the distribution of burned area in any given year. Model results indicate a combination of variables representing climate, topography, human presence and vegetation/fuel type best explain spatial variability in fire occurrence. Population density, growing season precipitation and slope were the most important predictors of the likelihood of fire occurrence followed by vegetation type and elevation. Key drivers of long-term means in fire activity were similar for the drier, North and the wetter, South bioclimatic zones but year-to-year variability in area burned and number of fires was higher in the more productive, fuel-rich South bioclimatic zone.

Matorral and plantation forests experience the highest mean annual area burned in the North bioclimatic zone and plantation forests, pasture lands and annual crops, and native deciduous forests in the South bioclimatic zone. Results from an evaluation of fire selective ‘preference’ for specific vegetation types highlight strong preference for exotic plantation forests, native sclerophyll forests, mixed native-exotic forests and matorral which together account for the highest proportion of fires burning in the North bioclimatic Zone. In the South bioclimatic zone resource selection analyses indicate strong fire preference for exotic plantation forests, mixed-native exotic forests and pasture lands and annual crops where high numbers of prescribed fires are used to burn crop stubble and manage pasture productivity. In contrast, results suggest strong fire ‘avoidance’ in agricultural and unvegetated lands in the North bioclimatic zone and native deciduous forests and unvegetated lands in the South bioclimatic zone. Strong fire avoidance in agricultural lands in the North bioclimatic zone is likely related to fire suppression aimed at protecting agricultural production.

Strong selective preference for fire occurrence in matorral, exotic plantation forests and dry sclerophyll forests more than would be expected in relation to their availability across the landscape can best be understood by examining the role that fuel structure, availability and condition contribute to fire occurrence and spread. All vegetation types where fire shows strong preference are characterized by abundant and connected fuels that are capable of promoting fire spread during warm, hot and dry conditions. Fire preference for exotic plantation and mixed native-exotic forests is likely a result of a combination of factors including: 1) the relatively homogenous and highly connected forest patch structure of exotic plantation forests compared to some native deciduous forests (primarily *Nothofagus* spp.), 2) abundant sources of accidental and intentional anthropogenic ignitions adjacent to exotic plantation forests, and 3) the fuel structure and fire-prone characteristics of *Eucalyptus* and *Pinus* plantations and native sclerophyllous forests [2, 60–62]. Recent research has documented increased fire activity in exotic plantations and suggests the combination of warming temperatures, increased plantation extent and changes in human ignitions are all playing some role [12, 19, 25, 63, 64]. Furthermore, fuel characteristics such as foliar flammability, fuel load, and plant structure of both native (e.g., *Diostea juncea*, *Lomatia hirsuta*) and nonnative (e.g., *Teline monspessulana*, *Acacia* spp.) plant taxa have been shown to facilitate fire spread and alter characteristics of fires that occur [24, 25, 65–67].

Patterns in the spatial distribution of burned area over the 2001–2017 period can be partially attributed to a warming and drying trend that played a role in the large fires that have occurred in Maule, Bío-Bío and Araucanía regions in 2016–2017 and China Muerta and Malleco National Reserves and Tolhuaca National Park in the Araucanía region in 2015 [12, 68]. Significant portions of these fires burned in native deciduous forests highlighting the fact that extreme fire weather can dry otherwise moist fuels sufficiently to allow for fire spread. Observations from over 350 rain stations in central Chile show annual rainfall has declined 7.9% since 1979 [5], whereas reanalysis data report declines in rainfall up to c. 30% per decade over the 1968–2001 time period in south-central Chile [69]. Additionally, central Chile experienced dramatic declines in rainfall (25–45%) between 2010–2015, a period termed the central Chile megadrought [7]. Declines in annual precipitation during the central Chile megadrought coincide with observed >1°C increases in annual mean temperatures and daily mean maximum temperatures (compared to 1970–2000 mean values) across inland valleys throughout central Chile [7, 70].

Our results lend further support to previous research that demonstrates interactions and feedbacks between non-native land cover and increasingly warm and dry conditions are combining to promote extensive fire activity. For example, the warm, biomass-rich lowland areas of the Bío-Bío and Araucanía regions that encompass extensive exotic forest plantations account for 60% of the total area burned within the study area each year even though they occupy only 46% of the land within the study area. Here the conversion of native forests to patches of highly flammable vegetation are likely exacerbating the effects of increasingly warmer and drier conditions on fire activity [8, 12]. Additionally, the abandonment of rural areas in some areas has led to increases in woody fuel accumulation and an overall decrease in fire control and suppression leading to increased fire occurrence [19, 71]. Lastly, the frequent use of fire as a management tool in pasture and annual croplands may be leading to fires that spread into adjacent forests, especially during years with exceptionally warm and dry conditions. These escaped fires have the potential to promote large fires in the normally moist, biomass-rich southern deciduous forests. Hence, the widespread replacement of native forests to more homogeneous, structurally continuous and fire-prone exotic plantation forests, and warming and drying trends are combining to further promote large fires throughout south-central Chile.

### The future of fire activity in south-central Chile

The large fires that have occurred in south-central Chile in recent years has sparked a national debate over the factors responsible and strategies for preventing and mitigating severe fires in the future. General frameworks used to predict fire activity in settings worldwide demonstrate that the intersection of patterns of climate, fuels and ignitions controls fire activity. Generally warmer temperatures, extended drying, the preponderance of well-connected and flammable vegetation and abundant anthropogenic ignitions are all factors linked to the occurrence of large fires in south-central Chile [12]. The interaction of a similar suite of factors was on dramatic display during the 2016–2017 fire season in Chile when over 580,000 hectares burned. Results from previous research and our analysis of recent fire activity suggest the combination of changing climatic conditions, land cover and land use will continue to promote large fire activity in south-central Chile [12].

First, climatic conditions and fire weather are known to influence the frequency, severity and size of wildfires. When extended periods of warm and dry conditions coincide with strong winds, small wildfires can rapidly grow to become large and severe, with dramatic impacts on vegetation and communities. Historically, climatic controls generally limited fire activity in south-central Chile to the dry summer season but fires are now occurring year-round. Climate models are largely in agreement that temperatures will continue to warm 3–4°C over the next several decades across central Chile [7, 70]. Concurrent with projections for warming temperatures, changes in several large-scale drivers of atmospheric circulation are expected to result in decreases in annual precipitation for much of south-central Chile. These include: 1) a continued positive phase of the Southern Annular Mode (SAM) which promote warm conditions in southern Chilean forests [59, 72] (such as the large 2016–2017 fire season), 2) the intensification of ocean-atmospheric teleconnections such as ENSO [7, 73] which result in strong swings between warm/wet and cool/dry years in central Chile [74], conditions which ultimately promote fuel development in wet years and fuel drying in warm, dry years, and 3) a negative trending Pacific Decadal Oscillation which directs storms entrained by southern westerly winds poleward, promoting long-term drought conditions in south-central Chile [74]. The interaction of these large-scale drivers amplified by anthropogenic climate change is predicted to lead to continued warming and drying such that by 2100 temperatures are expected to increase by up to 4°C and rainfall is projected to decline up to half of baseline (1960–1990) conditions [7, 75]. Hence, changing climatic conditions, namely warming temperatures and declining precipitation, are expected to result in conditions that promote large wildfires in south-central Chile into the future [12].

Second, the character and composition of vegetation across central Chile has altered the role that fuels play in regulating fire activity. Recent decades of land cover change has resulted in the shift from generally more structurally heterogeneous, fire-inhibiting forests to fire-prone systems exhibiting a more continuous fuel structure. Numerous studies investigating fuel flammability and structure show that vegetation that replaced once extensive deciduous forests provides more homogenous fuel loads that facilitate fire spread. Resource selection analyses of fire preference in south-central Chile provide evidence supporting this change, that the conversion of native forests to exotic plantations and pasture lands are changing fuels in ways that may further promote increased fire activity. Exotic plantation forests provide relatively homogenous, continuous and highly flammable fuels where fuels in native forests were historically more heterogeneous, less continuous and less flammable, especially in the wetter southern end of the study area.

Third, shifts in patterns of land-use that affect the spatial and temporal distribution of ignitions and fire suppression are also acting to influence fire activity in south-central Chile. Rural abandonment in some areas is leading to an accumulation of woody fuels where fuels were historically managed and fragmented. At the same time, the use of fires to manage pasture and agricultural lands may be leading to the accidental escape of deliberate fires into fuel-rich forests and exotic forest plantations. These fires and accidental ignitions associated with increases in population density and associated exurban development likely add to burned area. Intentional fires aimed at burning exotic forest plantations may also be responsible for large fires in biomass-rich forests, especially during extreme climatic conditions.

## Conclusions

Satellite derived fire data for south-central Chile show that the number of fires and their extent were highly variable in any given year. Our evaluation of the relationship between the spatiotemporal distribution of fires with climatic, topographic, human presence and land cover variables reveal several important patterns:

- Fire activity is closely tied to climatic (temperature and precipitation) and land cover (vegetation type) factors that influence the abundance, structure, continuity and condition of fuels and human factors (population density) that influences the distribution of ignitions.
- Drier areas of the North bioclimatic zone support fewer fires and lower mean annual area burned compared to the more productive, biomass-rich areas of the wetter South bioclimatic zone. Fuel-rich Araucanía and Bío-Bío regions account for 60% of all area burned across the study area even though these two areas account for only 46% of the entire study area.
- Matorral, pasture lands, exotic plantation forests and native sclerophyll forests represent a large proportion of the annual area burned in the North bioclimatic zone, likely a result of a mix of natural factors related to the spatial distribution of natural and human-caused ignitions and conditions driving fire spread (e.g., seasonality, temperature, wind, fuel moisture). These vegetation types all provide abundant, flammable and continuous fuels that support fire spread.
- Exotic plantation forests and pasture lands and annual crops represent the largest proportion of annual area burned in the wetter South bioclimatic zone. Exotic plantation forests provide abundant, structurally homogenous, connected and flammable fuels that can promote fire spread. The increase in the extent of plantation forests may interact with changing climatic conditions to promote large fires in south-central Chile. High mean annual area burned in pasture lands and annual crops is likely a result of the use of prescribed fires or “*quemas agrícolas*” to remove post-harvest agricultural and cereal crop stubble. Accidental spread of these fires may increasingly lead to large fires in adjacent, biomass-rich native and exotic plantation forests.
- Agricultural lands account for a low amount of annual area burned in the North bioclimatic zone compared to the availability of this vegetation type to burn. This is likely due to fire management aimed at protecting agricultural production and the fragmented distribution of fuels in these areas.
- Fire occurrence shows strong selective ‘preference’ for exotic plantation forests in both bioclimatic zones. This result highlights an important question—whether inherent flammability, land-use practices, natural, accidental or intentional ignitions or other factors are responsible for the disproportionately high occurrence of fire in plantation forests. The relative homogeneity and continuity of woody fuels associated with plantation forests may facilitate fire spread compared to other cover types where fuel structure is more heterogenous and discontinuous.
- Human activity in south-central Chile can either promote (through additional ignitions) or inhibit (through increased suppression effectiveness) fire activity. Our results suggest fire activity generally decreases with population density in both bioclimatic zones, however this relationship is weaker in the South bioclimatic zone where accidental/intentional fires in rural areas may contribute to fire occurrence and spread when extreme conditions promote fuel drying.
- During extreme fire conditions, hot, dry and windy conditions can lead to large fires in all vegetation types, including normally mesic deciduous forests that dominate the southern regions of the study area. Warming and drying trends that are projected to continue into the future for much of south-central Chile will further promote large fires in these fuel-rich forests that are often slow to recover from fires. This may result in a further decline in the abundance of native forest assemblages.

In summary, shifts in the fundamental controls on fire could portend a future with large increases in the frequency and extent of fires in south-central Chile. In light of recent large fires in Chile, efforts to reestablish more structurally and compositionally heterogeneous and less flammable native forests would likely buffer changes that are occurring in the climate system by reducing overall landscape flammability. With these anticipated changes, policymakers, managers and communities should prepare to accommodate more frequent and larger wildfires in the years to come. Policymakers and managers should also consider the role that fires in exotic plantation forests and pasture lands and annual crops may play in facilitating the spread of accidental fires into adjacent more fire-sensitive forests that may not readily recover following widespread fires. Clarifying the factors promoting large fires is becoming increasingly urgent as warmer temperatures and drier fire seasons provide conditions favorable for the occurrence of large fires throughout much of south-central Chile.

## Supporting information

S1 Fig Spatial distribution of the probability of fire occurrence based on best GLM. (EPS)

S2 Fig Fire selective ‘preference’ for specific vegetation types using R-package ‘Resource Selection Function’. (EPS)

S1 Table Pre-screening of variables for model comparison using importance values for variables included in final GLM and GAM model comparison. Highly correlated variables (Pearson’s correlation coefficient > 0.60) and variables with importance values < 0.05 were not included in model comparison.
(DOCX)

S2 Table Comparison of Random Forests variable importance values for the entire study area and the North and South bioclimatic zone models. Variables ranked according to their Random Forests variable importance values shown as the estimated mean decrease in accuracy (MDA).
(DOCX)

S3 Table Summary of GAM model comparison for study area and North and South bioclimatic zone model sets. Model AIC, delta AIC between best model and model shown, % deviance explained, model r-squared, degrees of freedom and predictor variables included in each model. All continuous variables were significant at the p < 0.0001 significance level.
(DOCX)

S1 File Image of high severity fire that occurred in an *Araucaria araucana* forest in 2015, China Muerta National Reserve, Araucanía region, south-central Chile (photo D. McWethy). (JPG)

## References

[1] Flannigan MD, Krawchuk MA, de Groot WJ, Wotton BM, Gowman LM. Implications of changing climate for global wildland fire. International Journal of Wildland Fire. 2009;18:483–507.

[2] Moreira F, Viedma O, Arianoutsou M, Curt T, Koutsias N, Rigolot E, et al Landscape—wildfire interactions in southern Europe: Implications for landscape management. Journal of Environmental Management. 2011;92(10):2389–402. 10.1016/j.jenvman.2011.06.028. 21741757

[3] Martinez-Harms MJ, Caceres H, Biggs D, Possingham HP. After Chile’s fires, reforest private land. Science. 2017;356(6334):147. 10.1126/science.aan0701 28408566

[4] Pausas J, Keeley J. Abrupt Climate-Independent Fire Regime Changes. Ecosystems. 2014:1–12. 10.1007/s10021-014-9773-5

[5] Boisier JP, Rondanelli R, Garreaud RD, Muñoz F. Anthropogenic and natural contributions to the Southeast Pacific precipitation decline and recent megadrought in central Chile. Geophysical Research Letters. 2016;43(1):413–21. 10.1002/2015GL067265

[6] Jolly WM, Cochrane MA, Freeborn PH, Holden ZA, Brown TJ, Williamson GJ, et al Climate-induced variations in global wildfire danger from 1979 to 2013. Nature Communications. 2015;6:7537 http://www.nature.com/articles/ncomms8537-supplementary-information. 26172867 10.1038/ncomms8537 PMC4803474

[7] Garreaud RD, Alvarez-Garreton C, Barichivich J, Boisier JP, Christie D, Galleguillos M, et al The 2010–2015 megadrought in central Chile: impacts on regional hydroclimate and vegetation. Hydrol Earth Syst Sci. 2017;21(12):6307–27. 10.5194/hess-21-6307-2017

[8] Miranda A, Altamirano A, Cayuela L, Lara A, González M. Native forest loss in the Chilean biodiversity hotspot: revealing the evidence. Regional Environmental Change. 2017;17(1):285–97. 10.1007/s10113-016-1010-7

[9] Batllori E, Parisien M-A, Krawchuk MA, Moritz MA. Climate change-induced shifts in fire for Mediterranean ecosystems. Global Ecology and Biogeography. 2013;22(10):1118–29. 10.1111/geb.12065

[10] Westerling AL, Hidalgo HG, Cayan DR, Swetnam TW. Warming and earlier spring increase western US forest wildfire activity. Science. 2006;313(5789):940–3. 10.1126/science.1128834 16825536

[11] Holz A, Veblen TT. Wildfire activity in rainforests in western Patagonia linked to the Southern Annular Mode. International Journal of Wildland Fire. 2012;21(2):114–26. 10.1071/Wf10121

[12] González ME, Gómez-González S, Lara A, Garreaud R, Díaz-Hormazábal I. The 2010–2015 Megadrought and its influence on the fire regime in central and south-central Chile. Ecosphere. 2018;In press.

[13] Úbeda X, Sarricolea P. Wildfires in Chile: A review. Global and Planetary Change. 2016;146:152–61. 10.1016/j.gloplacha.2016.10.004.

[14] CONAF. Análisis de la Afectación y Severidad de los Incendios Forestales ocurridos en enero y febrero de 2017 sobre los usos de suelo y los ecosistemas naturales presentes entre las regiones de Coquimbo y Los Ríos de Chile. Santiago, Chile: CONAF, 2017.

[15] Martínez R. Chile’s forest fires have been raging for weeks. What caused them? PRI. 2017 February 10, 2017.

[16] Watts J. Chile battles devastating wildfires: ‘We have never seen anything on this scale’. The Guardian. 2017.

[17] Thibaut F, Thomas C. Seasonal changes in the human alteration of fire regimes beyond the climate forcing. Environmental Research Letters. 2017;12(3):035006.

[18] García RA, Engler ML, Peña E, Pollnac FW, Pauchard A. Fuel characteristics of the invasive shrub Teline monspessulana (L.) K. Koch. International Journal of Wildland Fire. 2015;24(3):372–9.

[19] Carmona A, Gonzalez ME, Nahuelhual L, Silva J. Spatio-temporal effects of human drivers on fire danger in Mediterranean Chile. Bosque. 2012;33(3):321–8. 10.4067/S0717-92002012000300016

[20] Cóbar-Carranza AJ, García RA, Pauchard A, Peña E. Effect of Pinus contorta invasion on forest fuel properties and its potential implications on the fire regime of Araucaria araucana and Nothofagus antarctica forests. Biological Invasions. 2014;16(11):2273–91. 10.1007/s10530-014-0663-8

[21] Paritsis J, Landesmann BJ, Kitzberger T, Tiribelli F, Sasal Y, Quintero C, et al Pine Plantations and Invasion Alter Fuel Structure and Potential Fire Behavior in a Patagonian Forest-Steppe Ecotone. Forests. 2018;9(3). 10.3390/f9030117

[22] García RA, Pauchard A, Cavieres LA, Peña E, RodrÍguez MF. El fuego favorece la invasión de Teline monspessulana (Fabaceae) al aumentar su germinación. Revista chilena de historia natural. 2010;83:443–52.

[23] Blackhall M, Raffaele E, Veblen TT. Cattle affect early post-fire regeneration in a Nothofagus dombeyi–Austrocedrus chilensis mixed forest in northern Patagonia, Argentina. Biological Conservation. 2008;141(9):2251–61. 10.1016/j.biocon.2008.06.016.

[24] Pauchard A, Garcia RA, Pena E, Gonzalez C, Cavieres LA, Bustamante RO. Positive feedbacks between plant invasions and fire regimes: Teline monspessulana (L.) K. Koch (Fabaceae) in central Chile. Biological Invasions. 2008;10(4):547–53. 10.1007/S10530-007-9151-8

[25] Gomez-Gonzalez S, Torres-Diaz C, Valencia G, Torres-Morales P, Cavieres LA, Pausas JG. Anthropogenic fires increase alien and native annual species in the Chilean coastal matorral. Diversity and Distributions. 2011;17(1):58–67. 10.1111/J.1472-4642.2010.00728.X

[26] Nunez CI, Raffaele E, Nunez MA, Cuassolo F. When do nurse plants stop nursing? Temporal changes in water stress levels in Austrocedrus chilensis growing within and outside shrubs. Journal of Vegetation Science. 2009;20(6):1064–71.

[27] Franzese J, Raffaele E. Fire as a driver of pine invasions in the Southern Hemisphere: a review. Biological Invasions. 2017;19(8):2237–46. 10.1007/s10530-017-1435-z

[28] Echeverria C, Coomes D, Salas J, Rey-Benayas JM, Lara A, Newton A. Rapid deforestation and fragmentation of Chilean Temperate Forests. Biological Conservation. 2006;130(4):481–94. 10.1016/j.biocon.2006.01.017.

[29] Aguayo M, Pauchard A, Azocar G, Parra O. Land use change in the south central Chile at the end of the 20(th) century. Understanding the spatio-temporal dynamics of the landscape. Revista Chilena De Historia Natural. 2009;82(3):361–74.

[30] Giglio L, Loboda T, Roy DP, Quayle B, Justice CO. An active-fire based burned area mapping algorithm for the MODIS sensor. Remote Sensing of Environment. 2009;113(2):408–20. 10.1016/j.rse.2008.10.006.

[31] CONAF. Catastro de los Recursos Vegetacionales Nativos de Chile. In: Forestal CN, editor. Santiago, Chile 2017.

[32] Gajardo R. La Vegetación Natural de Chile: Clasificación y Distribución Gegráfica. Santiago, Chile: 1994.

[33] Veblen TT, Schlegel FM, Oltremari JV. Temperate broad-leaved evergreen forests of South America In: Ovington JD, editor. Temperate Broad-leaved Evergreen Forests. Amsterdam: Elsevier; 1983 p. 5–31.

[34] Luebert F, Pliscoff P. Bioclimatic and vegetative Chilean synopsis. House UP, editor. Santiago de Chile2006.

[35] Zhao M, Running SW. Drought-Induced Reduction in Global Terrestrial Net Primary Production from 2000 Through 2009. Science. 2010;329(5994):940–3. 10.1126/science.1192666 20724633

[36] Veblen TT, Young KR, Orme AR. The Physical Geography of South America. New York: Oxford University Press; 2007.

[37] Armesto JJ, Arroyo MTK, Hinojosa LF. The Mediterranean Environment of Central Chile In: Veblen TT, Young KR, Orme AR, editors. The Physical Geography of South America. Oxford: Oxford University Press; 2007 p. 360.

[38] Donoso P, Promis A. Forestry in Native Forests Advances in research in Chile, Argentina and New Zealand. Valdivia, Chile: Marisa Cuneo Ediciones; 2013.

[39] Amigo J, Ramírez C. A bioclimatic classification of Chile: woodland communities in the temperate zone. Plant Ecology. 1998;136(1):9–26. 10.1023/A:1009714201917

[40] Holz A, Haberle S, Veblen TT, De Pol-Holz R, Southon J. Fire history in western Patagonia from paired tree-ring fire-scar and charcoal records. Clim Past Discuss. 2012;7(5):3203–38. 10.5194/cpd-7-3203-2011

[41] Veblen TT. Temperate forests of the Southern Andean Region In: Veblen TT, Young KR, Orme AR, editors. The Physical Geography of South America. New York: Oxford University Press; 2007 p. 217–31.

[42] Veblen TT, Donoso C, Kitzberger T, Rebertus AJ. Ecology of southern Chilean and Argentinean Nothofagus forests In: Veblen TT, Hill RS, Read J, editors. Ecology and Biogeography of Nothofagus Forests: Yale University Press; 1996 p. 293–353.

[43] González ME, Cortés M, Izquierdo F, Gallo L, Echeverría C, Bekessy S, et al. Araucaria araucana. In: Donoso C, editor. Las especies arbóreas de los bosques templados de Chile y Argentina, Autoecología: Editorial Marisa Cuneo; 2013. p. 36–53.

[44] Holz A, Kitzberger T, Paritsis J, Veblen TT. Ecological and climatic controls of modern wildfire activity patterns across southwestern South America. Ecosphere. 2012;3(11). 10.1890/Es12-00234.1

[45] CONAF. Corporación Nacional Forestal of Chile Fire Dataset. In: Chile CNFo, editor. Santiago, Chile 2017.

[46] Humber ML, Boschetti L, Giglio L, Justice CO. Spatial and temporal intercomparison of four global burned area products. International Journal of Digital Earth. 2018:1–25. 10.1080/17538947.2018.1433727 PMC6178238 30319711

[47] Venables WN, Dichmont CM. GLMs, GAMs and GLMMs: an overview of theory for applications in fisheries research. Fisheries Research. 2004;70(2):319–37. 10.1016/j.fishres.2004.08.011.

[48] Breiman L. Random Forests. Machine Learning. 2001;45(1):5–32. 10.1023/A:1010933404324

[49] Team RC. R: a Language and Environment for Statistical Computing. R Foundation for Statistical Computing. Vienna, Austria 2016.

[50] Akaike H. Canonical Correlation Analysis of Time Series and the Use of an Information Criterion In: Mehra RK, Lainiotis DG, editors. Mathematics in Science and Engineering. 126: Elsevier; 1976 p. 27–96.

[51] S. K. Information Theory and Statistics: John Wiley & Sons; 1959.

[52] Shannon CE, Weaver W. The Mathematical Theory of Communication: Univ of Illinois Press; 1949.

[53] Hastie T, Tibshirani R, Friedman J. Elements of Statistical Learning. Second Edition ed: Springer; 1986 2009.

[54] Archer KJ, Kimes RV. Empirical charcterization of random forest variable importance measures. Computational Statistics & Data Analysis. 2008;52:2249–60.

[55] Sólymos P, Lele Subhash R. Revisiting resource selection probability functions and single-visit methods: clarification and extensions. Methods in Ecology and Evolution. 2015;7(2):196–205. 10.1111/2041-210X.12432

[56] Lele SR. A New Method for Estimation of Resource Selection Probability Function. Journal of Wildlife Management. 2009;73(1):122–7. 10.2193/2007-535

[57] Lele Subhash R, Keim Jonah L. Weighted distributions and estimation of resource selection probability functions. Ecology. 2006;87(12):3021–8. 10.1890/0012-9658(2006)87[3021:WDAEOR]2.0.CO;2 17249227

[58] Krebs CJ. Ecological Methodology. US: Pearson Education; 1998 624 p.

[59] Urrutia-Jalabert R, González Mauro E, González‐Reyes Á, Lara A, Garreaud R. Climate variability and forest fires in central and south-central Chile. Ecosphere. 2018;9(4):e02171 10.1002/ecs2.2171

[60] Viedma O, Angeler DG, Moreno JM. Landscape structural features control fire size in a Mediterranean forested area of central Spain. International Journal of Wildland Fire. 2009;18(5):575–83.

[61] Moreira F, Vaz P, Catry F, Silva JS. Regional variations in wildfire susceptibility of land-cover types in Portugal: implications for landscape management to minimize fire hazard. International Journal of Wildland Fire. 2009;18(5):563–74.

[62] Lara A, Zamorano C, Miranda A, González ME, Reyes R. Bosque Nativo In: Gligo N, editor. Informe País Estado del Medio Ambiente en Chile: comparación 1999–2015. Santiago, Chile: Centro de Análisis de Políticas Públicas, Universidad de Chile; 2016 p. 604.

[63] Diaz-Hormazabal I, Gonzalez ME. Spatio-temporal analyses of wildfires in the region of Maule, Chile. Bosque. 2016;37:147–58.

[64] Gonzalez ME, Lara A, Urrutia R, Bosnich J. Climatic change and its potential impact on forest fire occurrence in south-central Chile (33 degrees-42 degrees S). Bosque. 2011;32(3):215–9. 10.4067/S0717-92002011000300002

[65] Garcia RA, Pauchard A, Cavieres LA, Pena E, Rodriguez MF. Fire promotes Teline monspessulana (Fabaceae) invasion by increasing its germination. Revista Chilena De Historia Natural. 2010;83(3):443–52.

[66] Blackhall M, Raffaele E, Veblen TT. Is foliar flammability of woody species related to time since fire and herbivory in northwest Patagonia, Argentina? Journal of Vegetation Science. 2012;23(5):931–41. 10.1111/j.1654-1103.2012.01405.x

[67] Blackhall M, Veblen TT, Raffaele E. Recent fire and cattle herbivory enhance plant-level fuel flammability in shrublands. Journal of Vegetation Science. 2015;26(1):123–33. 10.1111/jvs.12216

[68] González ME, Lara A. Large fires in the Andean Araucaria forests: when a natural ecological process becomes a threat. Oryx. 2015;49(3):394-. Epub 07/01. 10.1017/S0030605315000599

[69] Garreaud R, Lopez P, Minvielle M, Rojas M. Large-Scale Control on the Patagonian Climate. Journal of Climate. 2012;26(1):215–30. 10.1175/JCLI-D-12-00001.1

[70] IPCC. Climate Change 2014: Synthesis Report. Contribution of Working Groups I, II and III to the Fifth Assessment Report of the Intergovernmental Panel on Climate Change. Pachauri RK, Meyer LA, editors. Geneva, Switzerland: IPCC 2014. 151 p.

[71] Viedma O, Moity N, Moreno JM. Changes in landscape fire-hazard during the second half of the 20th century: Agriculture abandonment and the changing role of driving factors. Agriculture, Ecosystems & Environment. 2015;207(Supplement C):126–40. 10.1016/j.agee.2015.04.011.

[72] Holz A, Paritsis J, Mundo IA, Veblen TT, Kitzberger T, Williamson GJ, et al Southern Annular Mode drives multicentury wildfire activity in southern South America. Proceedings of the National Academy of Sciences. 2017;114(36):9552–7. 10.1073/pnas.1705168114 PMC5594661 28827329

[73] Cai W, Santoso A, Wang G, Yeh S-W, An S-I, Cobb KM, et al ENSO and greenhouse warming. Nature Clim Change. 2015;5(9):849–59. 10.1038/nclimate2743

[74] Garreaud RD, Vuille M, Compagnucci R, Marengo J. Present-day South American climate. Palaeogeography, Palaeoclimatology, Palaeoecology. 2009;281(3):180–95. 10.1016/j.palaeo.2007.10.032.

[75] Fuenzalida H AP, Falvey M, Garreaud R, Rojas M, Sanchez R. Study on climate variability for Chile during the 21st century. Santiago, Chile: 2007.

