# Correction: Landscape drivers of recent fire activity (2001-2017) in south-central Chile

**DOI:** 10.1371/journal.pone.0205287

**Published:** 2018-10-02

**Authors:** David B. McWethy, Aníbal Pauchard, Rafael A. García, Andrés Holz, Mauro E. González, Thomas T. Veblen, Julian Stahl, Bryce Currey

Fig 5 is incorrect. The authors have provided a corrected version here.

**Fig 5:**
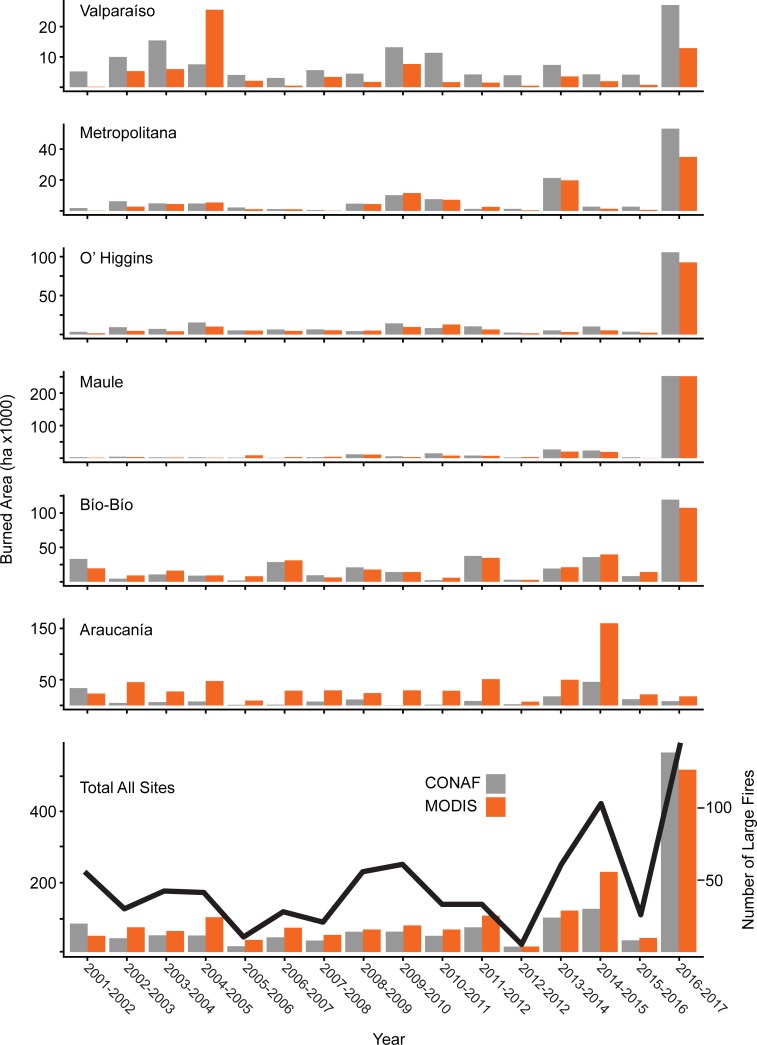
Total burned are for each region by year. Burned area by year across administrative regions based on CONAF dataset (gray bars) and MODIS Collection 6 burned area detections (orange bars; Y-axis scale varies for each region). Total burned area for all districts based on CONAF dataset and MODIS detected burned area (bottom panel). Total number of large fires (>200 ha) by year from CONAF dataset indicated by black line.
