# Supplementary material for: Landscape drivers of recent fire activity (2001-2017) in south-central Chile
Source: PLoS One. 2018 Aug 22;13(8):e0201195. doi: 10.1371/journal.pone.0201195 (PMC6104937; doi:10.1371/journal.pone.0201195)
Supplement: S1 Table — Highly correlated variables (Pearson’s correlation coefficient > 0.60) and variables with importance values < 0.05 were not included in model comparison. (DOCX) [file pone.0201195.s003.docx]

**S1 Table. Pre-screening of variables for model comparison using Importance Values for variables included in final GLM and GAM model comparison.** Highly correlated variables (Pearson’s correlation coefficient > 0.60) and variables with importance values < 0.05 were not included in model comparison.

| **Study Area** | **Imp. Val.** | **North BZ** | **Imp. Val.** | **South BZ** | **Imp. Val.** |
| --- | --- | --- | --- | --- | --- |
| Elevation | 0.53 | Vegetation Type | 0.48 | Elevation | 0.48 |
| Vegetation Type | 0.52 | Slope | 0.30 | Grow Seas. Precip. | 0.46 |
| Population Density | 0.27 | Grow Seas. Precip. | 0.24 | Population Density | 0.26 |
| Grow Seas. Precip. | 0.23 | Population Density | 0.18 | Vegetation Type | 0.26 |
| Slope | 0.23 | Elevation | 0.15 | Slope | 0.11 |
