# Supplementary material for: Landscape drivers of recent fire activity (2001-2017) in south-central Chile
Source: PLoS One. 2018 Aug 22;13(8):e0201195. doi: 10.1371/journal.pone.0201195 (PMC6104937; doi:10.1371/journal.pone.0201195)
Supplement: S3 Table — Model AIC, delta AIC between best model and model shown, % deviance explained, model r-squared, degrees of freedom and predictor variables included in each model. All continuous variables were significant at the p < 0.0001 significance level. (DOCX) [file pone.0201195.s005.docx]

**S3 Table. Summary of GAM model comparison for Study Area and North and South bioclimatic zone model sets.**  Model AIC, delta AIC between best model and model shown, % deviance explained, model r-squared, degrees of freedom and predictor variables included in each model. All continuous variables were significant at the p < 0.0001 significance level.

| **Study Area** |  |  |  |  |  |
| --- | --- | --- | --- | --- | --- |
| **AIC** | **ΔAICc** | **% Dev. Exp.** | **R-sq.** | **DF** | **Predictor Variables** |
| 35929 | 0.0 | 12.4 | 0.830 | 41 | elevation, vegetation type, population density, growing season precipitation, slope |
| 35973 | 44 | 12.3 | 0.082 | 33 | elevation, vegetation type, population density, growing season precipitation |
| 36874 | 945 | 10.1 | 0.060 | 25 | elevation, vegetation type, population density |
| 37020 | 1090 | 9.68 | 0.058 | 17 | elevation, vegetation type |
| 37020 | 1090 | 6.65 | 0.369 | 17 | elevation |
| 41133 | 5203 | <0.0001 | <0.0001 | 1 | null model |
|  |  |  |  |  |  |
| **North Bioclimatic Zone** | | |  |  |  |
| **AIC** | **ΔAICc** | **% Dev. Exp.** | **R-sq.** | **DF** | **Predictor Variables** |
| 14690 | 0.0 | 12.6 | 0.089 | 39 | vegetation type, slope, growing season precipitation, population density, elevation |
| 15036 | 346 | 10.5 | 0.070 | 30 | vegetation type, slope, growing season precipitation, population density |
| 15127 | 437 | 9.9 | 0.680 | 25 | vegetation type, slope, growing season precipitation |
| 15431 | 741 | 7.98 | 0.055 | 16 | vegetation type, slope |
| 15787 | 1097 | 5.75 | 0.035 | 8 | vegetation type |
| 16773 | 2083 | <0.0001 | <0.0001 | 1 | null model |
|  |  |  |  |  |  |
| **South Bioclimatic Zone** | | |  |  |  |
| **AIC** | **ΔAICc** | **% Dev. Exp.** | **R-sq.** | **DF** | **Predictor Variables** |
| 18837 | 0.0 | 14.9 | 0.13 | 37 | elevation, growing season precipitation, population density, vegetation type, slope |
| 18884 | 47 | 14.6 | 0.13 | 32 | elevation, growing season precipitation, population density, vegetation type |
| 18946 | 109 | 14.3 | 0.127 | 25 | elevation, growing season precipitation, population density |
| 19394 | 557 | 12.2 | 0.098 | 18 | elevation, growing season precipitation |
| 20559 | 1721 | 6.85 | 0.046 | 9 | elevation |
| 22078 | 3241 | <0.0001 | <0.0001 | 1 | null model |
