# Supplementary figures and images for: Landscape drivers of recent fire activity (2001-2017) in south-central Chile
Source: PLoS One. 2018 Aug 22;13(8):e0201195. doi: 10.1371/journal.pone.0201195 (PMC6104937; doi:10.1371/journal.pone.0201195)

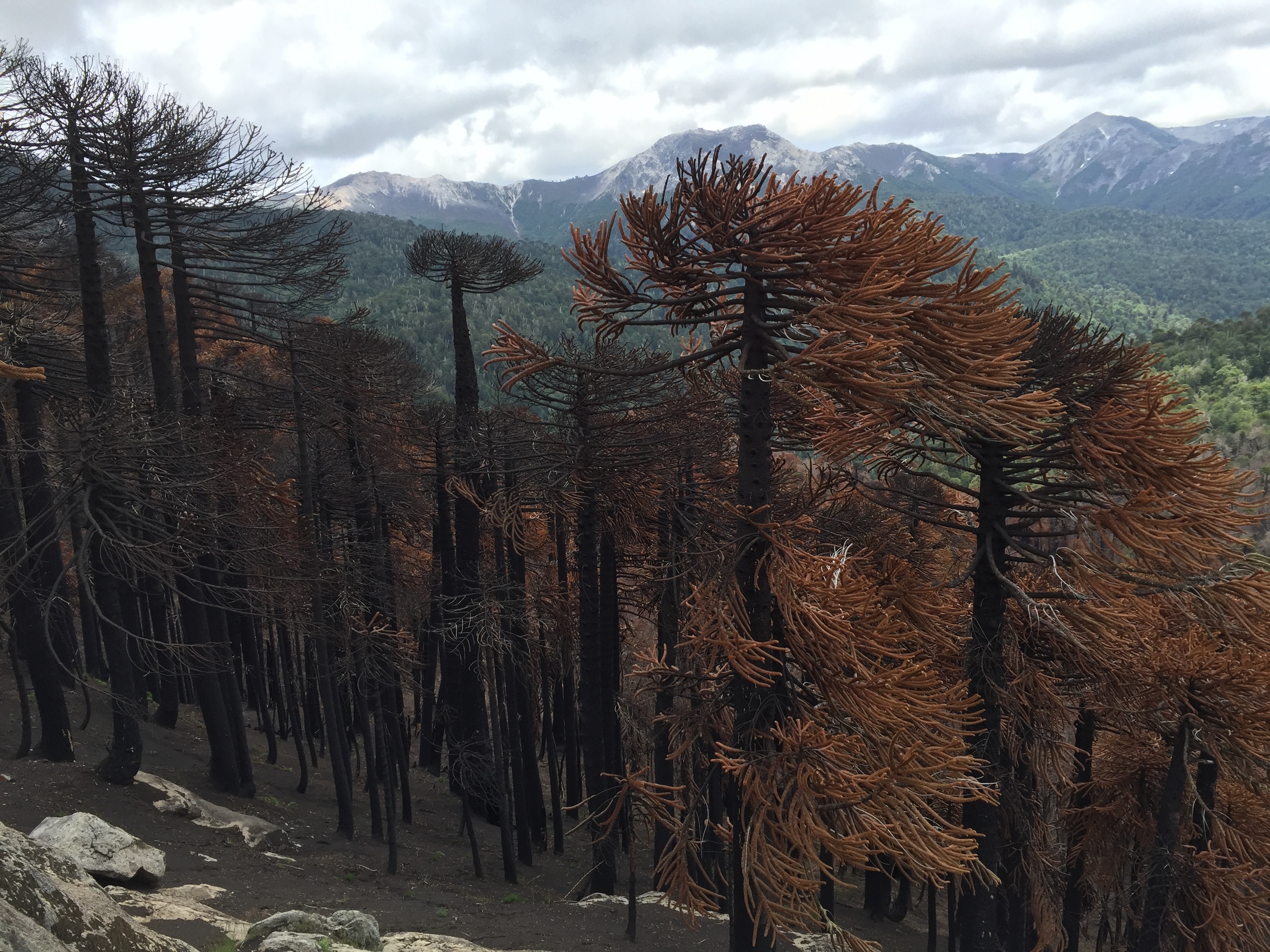

Supplement: S1 File — (JPG) [file pone.0201195.s006.jpg]
